# Factors influencing perceptions of private water quality in North America: a systematic review

**DOI:** 10.1186/s13643-019-1013-9

**Published:** 2019-05-10

**Authors:** Abraham Munene, David C. Hall

**Affiliations:** Department of Ecosystem and Public Health, Faculty of Veterinary Medicine, University of Calgary, 3280 Hospital Drive NW, Calgary, AB T2N 4Z6 Canada

**Keywords:** Private water, Water wells, Perceptions, Well stewardship, Public health

## Abstract

**Background:**

An estimated four million and 43 million people in Canada and the USA use private water supplies. Private water supplies are vulnerable to waterborne disease outbreaks. Private water supplies in Canada and the USA are often unregulated and private water management is often a choice left to the owner. Perceptions of water quality become important in influencing the adoption of private water stewardship practices, therefore safeguarding public health.

**Methods:**

We conducted a systematic literature review to understand factors that shape perceptions of water quality among private water users. We searched six computer databases (Web of science, Medline, Scopus, EBSCO, PubMed and Agricola). The search was limited to primary peer-reviewed publications, grey literature and excluded conference proceedings, review articles, and non-peer review articles. We restricted the search to papers published in English and to articles which published data on surveys of private water users within Canada and the USA. The search was also restricted to publications from 1986 to 2017. The literature search generated 36,478 records. Two hundred and four full text were reviewed.

**Results:**

Fifty-two articles were included in the final review. Several factors were found to influence perceptions of water quality including organoleptic preferences, chemical and microbiological contaminants, perceived risks, water well infrastructure, past experience with water quality, external information, demographics, in addition to the values, attitudes, and beliefs held by well owners.

**Conclusions:**

Understanding the factors that shape perceptions of water quality among private water users is an important step in developing private water management policies to increase compliance towards water testing and treatment in Canada and the USA. As many jurisdictions in Canada and the USA do not have mandatory private water testing or treatment guidelines, delineating these factors is an important step in informing future research and guiding policy on the public health of private water systems.

**Electronic supplementary material:**

The online version of this article (10.1186/s13643-019-1013-9) contains supplementary material, which is available to authorized users.

## Introduction

An estimated four million and 43 million people in Canada and the USA use private water supplies [1, 2]. In the absence of municipal water distribution systems in rural populations, private water supplies are an alternative source of domestic water in developed countries. Private water supplies are vulnerable to waterborne disease outbreaks [3, 4]. Several chemical and microbiological contaminants can contaminate private water supplies. Nutrients (e.g. nitrates), pathogens, pharmaceuticals, hormones, heavy metals, nanomaterials and personal care products are some contaminants that have been identified in well water [5–10]. These contaminants are associated with illnesses including gastrointestinal illnesses, liver and kidney problems, endocrine disruption, cancer, reproductive issues and neurological disorders [11].

Private water supplies in Canada and the USA are often unregulated. Management of private water supplies (e.g. water wells, cisterns or boreholes) is the responsibility of the owner. As a guide to drinking water quality standards, the Guidelines for Canadian Drinking Water Quality set out national drinking water standards in Canada. Similarly, the Safe Water Drinking Act is used to set out national and enforceable drinking water guidelines in the USA. However, the legislation excludes private water sources that serve less than 25 people. Private water systems are defined by water systems that serve 25 people or less for at least 60 days within a year and have up to 15 service connections. Water wells make up the majority of private water systems with cisterns and residential wells also considered as private water systems [12]. Approximately four million and over 13 million people are estimated to rely on unregulated private water wells in Canada and the USA [13, 14]. Although both Canada and the USA have national guidelines for the minimum standards of drinking water quality, there may be jurisdictional differences in the contaminants that are assessed [15]. Furthermore, individual provinces or states may have their own regulations on the construction of new wells, how many service connections can be served by a private water supply, and water testing recommendations, with some provinces or states requiring mandatory testing of wells upon the acquisition of new properties [12, 16]. Unlike municipal water supplies which may be regularly monitored and treated, few regulations cater to testing and treatment of private water supplies in Canada and the USA [1, 16–18].

In the absence of regulations on the management of private water supplies, compliance to private water testing and treatment becomes an essential mitigation strategy in protecting the health of private water users from diseases that could be contracted from consuming contaminated water. Recent studies indicate that compliance towards private water testing and treatment recommendations in various jurisdictions is low [2, 19]. Roche et al. (2013) found that nearly 80% of respondents in their survey tested water quality at frequencies below the current provincial recommendations. Perceptions of water quality may influence the adoption and implementation of private water management practices [20]. The choice of when to test water quality, what to test for, and what treatment devices to use on private water systems are decisions that are based on both perception and knowledge of risks to private water contamination.

Perceptions have been broadly defined as a human being’s primary cognitive contact with the environment or simply the way in which we understand the world around us using our senses [21]. However, this narrow definition based on the sensory appraisal we make to understand our environment is myopic and does not capture the complexity of factors involved in shaping perceptions. Perception also has subjective components that are associated with learning and past experiences that are mediated by attention, memory, and the ability to retrieve information from memory [22].

Consequently, this raises the question; what factors are important in shaping the perceptions private water users have of their water quality? Little is known about the factors that influence perceptions of water quality among private water users. We conducted a systematic review of studies on people reliant on private water systems for domestic use in both Canada and the USA to determine the factors that influence the perceptions of water quality within these two countries. Describing and understanding the factors that shape perceptions of water quality among private water users is an important step in developing well water management policies to increase compliance towards private water stewardship practices such as water testing and treatment in Canada and the USA. To guide the scope of our systematic review, we wanted to answer the main question. What factors predominantly drive perceptions of private water quality in Canada and the USA?

## Methods

### PICO framework

A PICO framework [23] was used to help guide the questions of the review. As most studies included were observational, assessment for control groups was not feasible as there would be no adequate comparison for perceptions held by private water users to a similar group (Table 1).

**Table 1:** PICO framework

PICO	Characteristic assessed
Population of interest	Studies reporting on private water users within Canada and the USA^a^
Intervention	Factors influencing perception of water quality
Control	Not applicable
Outcome	Presence of water treatment and water testing

^a^Some studies that reported on both private and municipal supplies were included in the review

### Search strategy

Literature searches were made on both health and environmental databases. A search strategy was developed in consultation with a research librarian and the review team. Our review was informed by methods for conducting systematic reviews in agri-food research [24]. We searched six computer databases (Web of science, Medline, Scopus, EBSCO, PubMed, and Agricola). The search was conducted between January and December 2017. The search was limited to primary peer-reviewed publications and grey literature. The search excluded conference proceedings, review articles, and non-peer review articles. We restricted the search to articles published in English and to articles which published data on private water users within Canada and the USA. The search was restricted to publications within the last 31 years (01/01/1986–31/12/2017). This time frame was used to capture recent amendments in regulations within the Safe Water Drinking Act and the Guidelines for Canadian Drinking Water Quality which may influence what substances are considered as drinking water contaminants and at what maximum acceptable concentration (MAC). A combination of search phrases was used for each database but consisted of major search domains with associated synonyms required to capture relevant articles. Keywords searched were *private water*, *domestic water*, *household water*, *well water*, *drinking water*, *perceptions*, *knowledge*, *belief*, *attitude*, *information*, *awareness*, *testing*, *treatment*, *survey,* and *rural*. Reference lists for relevant primary articles and review articles were screened. Articles fitting the inclusion criteria, that is, articles that were published in English, articles that conducted surveys on human participants relying on private water sources through questionnaires or interviews, articles that surveyed participants in Canada or the USA, articles that were primary research and articles that had the outcomes of private water testing, treatment or investigate alternative water use in the context of private water users were added to the final list. All study approaches were considered including quantitative, qualitative, and mixed methods. As the focus was on perceptions of water quality among private water users, studies that directly surveyed private water owners were included in the final literature search (Table 2 with key terms used and the number of papers generated for each phrase search is provided. See Additional file 1: Table S1).

**Table 2:** Factors identified to influence perceptions of private water quality with associated studies

Article	Year published	Location	Sample size	Study approach	Factors discussed
Jones et al. [1]	2006	Hamilton (CA)	246	Quantitative	Well infrastructure, demographic factors, organoleptic properties, chemical and microbiological contaminants, external information
Flanagan et al. [2]	2015	Maine (USA)	386	Quantitative	Demographic factors, perceived risk, chemical contaminants, organoleptic properties, well infrastructure
Jones et al. [17]	2005	Hamilton (CA)	16	Qualitative	Organoleptic properties, perceived risk, external information
Flanagan et al. [18]	2015	Maine (USA)	525	Quantitative	Chemical and microbiological contaminants, demographic factors, values, attitudes, and beliefs, well infrastructure
Roche et al. [19]	2013	Newfoundland and Labrador (CA)	618	Quantitative	Demographic factors, well infrastructure, organoleptic properties, external information
Garcia et al. [26]	2016	Texas, Arizona and New Mexico (USA)	47	Quantitative	Demographic factors, organoleptic properties, chemical and microbiological contaminants, past experience
Murti et al. [27]	2016	Arkansas, Indiana and Oklahoma (USA)	41	Qualitative	Chemical and microbiological contaminants, organoleptic properties, external information
Colt et al. [28]	2002	New Hampshire (USA)	98	Quantitative	Chemical contaminants, well infrastructure
Shaw et al. [29]	2005	Churchill county, Nevada (USA)	351	Quantitative	Chemical contaminants, perceived risk, demographic factors
Schwartz et al. [30]	1998	New York (USA)	244	Quantitative	Demographic factors, well infrastructure, perceived risk, chemical and microbiological contaminants, organoleptic properties
Poe et al. [31]	1998	Wisconsin and New York (USA)	307	Quantitative	Chemical contaminants, perceived risk
Lewandowski et al. [32]	2008	Minnesota (USA)	483 surveys 377 testing kits	Quantitative	Well infrastructure, chemical and microbiological contaminants, organoleptic properties
Pieper et al. [33]	2015	Virginia (USA)	2146	Quantitative	Chemical and microbiological contaminants, organoleptic properties, well infrastructure
Postma et al. [34]	2011	Gallatin County (USA)	188 households (320 children)	Quantitative	Demographic factors, chemical and microbiological contaminants
Mechenich et al. [35]	1994	Wisconsin (USA)	139	Quantitative	Chemical contaminants, attitudes, and perceived risk
Strauss et al. [36]	2001	Ontario (CA)	647	Quantitative	Demographic factors, microbiological contaminants
Schade et al. [37]	2015	West Virginia (USA)	498	Quantitative	External information, chemical contaminants
Walker et al. [39]	2006	Churchill county Nevada (USA)	351	Quantitative	Chemical contaminants, perceived risk,
McLeod et al. [40]	2014	Saskatchewan (CA)	1294	Quantitative	Demographic factors, external information, values, attitudes, and beliefs
McLeod et al. [41]	2015	Saskatchewan (CA)	1294	Quantitative	Organoleptic properties, past experience
Levallois et al. [42]	1998	Quebec (CA)	222	Quantitative	Organoleptic properties, chemical contaminants, well infrastructure
Acharya et al [43]	2008	Alberta (CA)	33	Quantitative	Organoleptic properties, perceived risk, microbiological contaminants
McSpirit et al. [44]	2011	West Virginia (US)	256	Quantitative	Demographic factors, organoleptic properties, perceived risk
Merkel et al. [45]	2012	Pennsylvania (USA)	158	Mixed methods	Organoleptic characteristics, demographic factors, perceived risk, values, attitudes, and beliefs
Summers [46]	2010	Alberta (CA)	1014	Quantitative	Demographic factors, well infrastructure, values, attitudes, and beliefs, organoleptic properties, chemical and microbiological contaminants
Chappells et al. [47]	2015	Nova Scotia (CA)	420 (32 in depth interviews)	Mixed methods	Demographic factors, perceived risk, organoleptic properties, chemical and microbiological contaminants, past experience
Flanagan et al. [49]	2016	Maine and New Jersey (USA)	344	Quantitative	Chemical contaminant, values, attitudes, and beliefs
Straub and Leahy [50]	2014	New England, Connecticut, Rhode Island, Maine, New Hampshire and Vermont (USA)	513/776 for children and 452/776 for parent	Quantitative	Demographic factors, organoleptic properties, perceived risk, external information
Lothorp et al. [51]	2016	Arizona (USA)	31/34	Quantitative	Demographic factors, chemical contaminants
Kreutzwiser et al. [52]	2011	Ontario (CA)	1567	Quantitative	Well infrastructure, microbiological contaminants, past experience, external information
Mahler et al. [48]	2014	Alaska, Idaho, Oregon, Washington (USA)	225	Quantitative	Organoleptic properties, demographic factors, perceived risk
Schubert et al. [53]	1999	Wisconsin (USA)	562	Quantitative	Demographic factors, chemical contaminants, external information
Feinman et al. [54]	2015	New Mexico (USA)	6606	Quantitative	Demographic factors
Imgrund et al. [55]	2011	Ontario (CA)	22	Qualitative	Perceived risk, well infrastructure, values, attitudes, and beliefs. microbiological contaminants
Jones et al. [56]	2007	British Columbia (CA)	4612	Quantitative	Demographic factors, perceived risks, microbiological contaminants
Johnson [57]	2008	New Jersey	266	Quantitative	Demographic factors, values, attitudes, and beliefs, past experience
Renaud et al. [58]	2011	Quebec (CA)	542	Quantitative	Demographic factors, external information, chemical contaminants
Ridpath et al. [59]	2016	48 states within the USA	1100	Quantitative	Chemical and biological contaminants, external information, well infrastructure
Flanagan et al. [60]	2016	New Jersey (USA)	711	Quantitative	Demographic factors, external information, perceived risk, chemical contaminants
Flanagan et al. [62]	2016	New Jersey (USA)	670	Quantitative	Demographic factors, values, attitudes, and beliefs, perceived risk, chemical contaminants
Laflamme et al. [66]	2004	Washington (USA)	6927	Quantitative	Perceived risk, chemical contaminants
Severtson et al. [67]	2006	Wisconsin (USA)	545	Quantitative	Demographic factors, perceived risk, chemical contaminants, past experience
Severtson et al. [68]	2008	Wisconsin (USA)	897	Mixed methods	Chemical contaminants, demographic factors, perceived risk
Slotnick et al. [69]	2006	Michigan (USA)	221	Quantitative	Chemical contaminants, well infrastructure
Kite-Powell et al. [70]	2006	Oregon (USA)	102	Quantitative	Chemical contaminants
Tabbot [71]	2006	New Jersey (USA)	50	Quantitative	Chemical and microbiological contaminants, organoleptic properties
Hexemer et al. [72]	2008	Ontario (CA)	248	Quantitative	Chemical and microbiological contaminants, demographic factors, external information
Swistock et al. [73]	2012	Pennsylvania (USA)	450	Quantitative	Chemical and microbiological contaminants, well infrastructure
Paul et al. [74]	2015	Tuftonboro (USA)	285	Quantitative	External information
Pintar et al. [75]	2009	Ontario (CA)	2332	Quantitative	Demographic factors
Yu et al. [76]	2014	Nova Scotia (CA)	960	Quantitative	Demographic factors, well infrastructure, chemical contaminants
Malecki et al. [77]	2017	Wisconsin (USA)	460	Quantitative	Organoleptic properties, demographic factors, chemical and microbiological contaminants

### Data extraction

Each paper included in the final review was read independently by the two authors and then assessed for relevancy in the review. The lead author extracted the following information: the main purpose of the study, the study population, study approach, methods of data collection, whether theoretical frameworks were used in the study, notes on the context of use of the private water systems, whether a formal intervention was present and results. The author also constructed a table identifying the study type, demographics, the intervention being evaluated and results of relevance to the present study (Additional file 2: Table S2). The second author independently verified data extraction and tabulation for the included articles. Each article included in the final list was independently rated by both authors for relevance to the review. Both authors met regularly over a period of 4 months to discuss the findings. In instances of disagreement, articles were reassessed independently, and consensus was reached following deliberation and discussion by the authors. A PRISMA flow diagram was used to narrow our selection of articles [25]. Articles were preliminarily screened by (1) reviewing the article titles generated by the keywords search, (2) reviewing article abstracts, (3) reviewing the full articles, and (4) sorting on relevance for the review.

### Quality of study and risk of bias

As a measure of the quality of study, articles were evaluated by whether they were published in a peer reviewed journal (as the assumption is that articles published in reviewed journals have been adequately scrutinised by reviewers before publication) or were technical reports. Reviewers also ranked the quality of the study relative to the review’s objectives on a scale. A risk of bias assessment from each study was conducted using the Strobe checklist assessment for risk of bias. Studies were ranked on a scale of 1 (high quality) to 4 (low quality) for their relevance to the review and based on the strobe checklist.

## Results

**Fig. 1:**
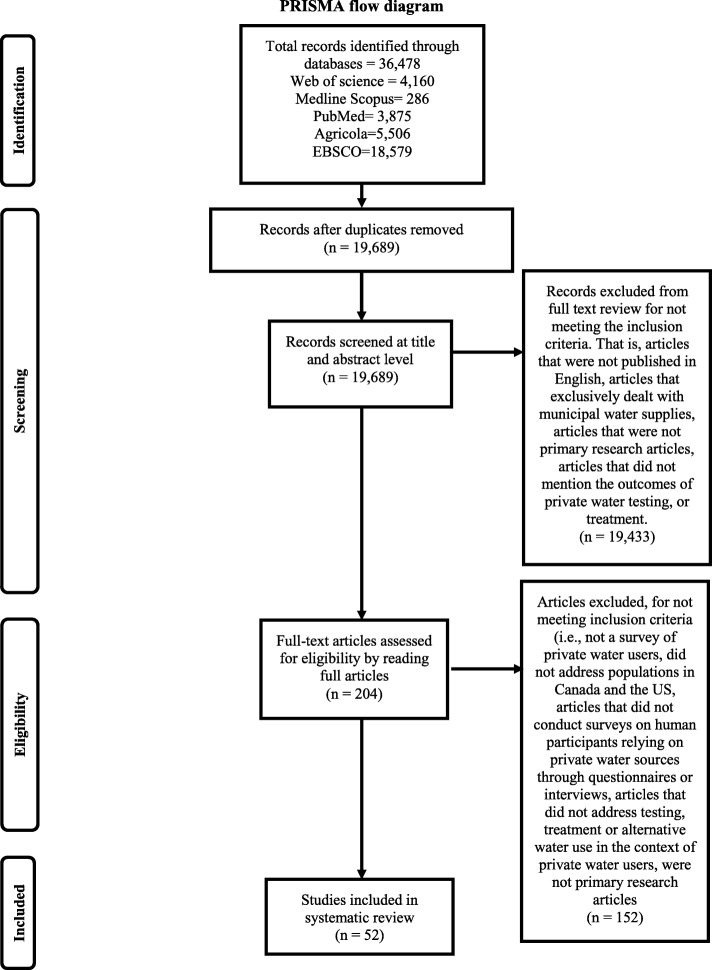
PRISMA flow diagram for study selection

The database search included 36,478 articles using the keyword search. Web of Science (*n* = 4160), Medline OVID (*n* = 286), Scopus (*n* = 3875), PubMed (*n* = 4072), Agricola (*n* = 5506), EBSCO (*n* = 18,579). Ultimately, 204 papers were examined intensively of which 152 articles were excluded for not meeting the relevance criteria for this study (Fig. 1). Fifty-two studies were included in the final review. Of the 52 studies identified, 44 exclusively focused on surveys delivered to private water supply owners while ten studies surveyed both residents with private and municipal supplies. Most of the articles (*n* = 35) were from the USA while 17 articles reported on private water users in Canada. All studies were observational. Most of the studies used a cross-sectional design (*n* = 49) with the rest reporting on case control studies. Studies were also classified as quantitative (*n* = 48), mixed methods (*n* = 3) or qualitative (*n* = 3). Survey administration methods varied. Questionnaire mail deliveries were used in 35 out of 52 studies and telephone surveys were used in 11 out of 52 studies. Other methods used to elicit participation included face to face interviews (3 out of 52) and focus groups (6 out of 52).

This systematic review included 52 journal articles with data collected on over 35,000 well water owners across Canada (*n* = 14,793) and the USA (*n* = 22,420). Perceptions of well water quality across Canada and the USA were found to be influenced by several factors. The main factors identified through this review were organoleptic properties of water, knowledge of chemical and microbiological contaminants, perceived risk, demographic factors, past experience with water quality, external information, values, attitudes, and beliefs about water, and water infrastructure.

### Organoleptic properties of water

Private water owners primarily relied on the sensory properties of drinking water sourced from their wells when it came to decisions regarding well management options. Decisions on when to test water quality or the choice to consume water were often instigated by changes in either the taste, look or smell of the water [1, 2, 17–19]. Satisfaction with the organoleptic properties of water was not necessarily equated to concern over drinking water sourced from the wells. For example, although most respondents rated the organoleptic properties of their water from ‘good’ to ‘very good’, nearly 80% of respondents to the survey indicated being concerned about their water quality [1]. In contrast, organoleptic properties of drinking water were congruent with the perceptions of the safety of water for consumption. About 67% of participants who had issues with the organoleptic properties did not consider their water as safe to consume [26]. Sensory cues derived from the organoleptic properties of water were not only limited to water consumed but also to other water uses. For example, some people reported on the hardness of their well water as it tended to discolour their appliances or plumbing systems [17]. Due to psychological factors, people expect sensorial information on the taste odour, and colour of water to be congruent [20]. However, what is not clear from the studies is what sense dominated when well water owners indicated a change in their well water quality. Evidence on how well water owners perceive the taste, smell, and odour of water sourced from their wells relative to alternative water sources such as bottled water or municipal tap water was also evaluated in some studies. Well water owners were unwilling to change to municipal water supplies due to their personal preference for the taste of their well water and fear of ‘chemicals’ in city water [27]. Similarly, Jones et al. (2005) found that well water owners preferred their well water over bottled water due to preferences in taste and scepticism to where the bottled water came from.

### Chemical and microbiological contaminants

Due to the soluble properties of water, several chemical and microbiological substances can be found in private water sources. Some chemical and microbiological substances can pose a health risk to individuals consuming well water. Of the 52 articles included, 13 out of 52 exclusively focused on assessing exposure to naturally occurring arsenic. Nitrate exposure was exclusively evaluated in 5 out of 52 articles. Radon exposure was exclusively evaluated in 1 out of 52 articles while 2 out of 52 articles evaluated the exposure of *Escherichia coli* and total coliforms on well water. Thirty-four articles were non-specific towards the chemical or microbiological contaminants (e.g. general microbiological and chemical contamination or a combination of both). For studies exclusively focusing on exposure to one contaminant, some studies were clear about the thresholds for the MAC for contaminants. The MAC is the specific level of a contaminant that is allowed in water for a specific purpose (e.g., human consumption). The contaminants assessed for and the MAC for specific contaminants may vary regionally [15]. However, the MAC’s of several contaminants in Canada and the USA are similar and reflect standards set out by the US Environmental Protection Agency. Studies examining naturally occurring arsenic as an exposure often quoted 10 μg/l as the MAC [2, 28, 29]. For nitrate as an exposure, the MAC used was 10 mg/L in six studies [30–32]. Lead exposure was assessed in one of the studies [33] with some studies quoting MAC’s for each contaminant assessed [34]. However, in some of the studies that assessed multiple exposure to contaminants or that were non-specific to a contaminant, MAC’s were not used [26, 35–37].

Some studies also included a water testing component to evaluate the prevalence of contaminants of interest in their samples (Table 3).

**Table 3:** Prevalence of contaminants within well water in surveys of well water owners

Study	Contaminant	Proportion of participants exceeding MAC
Pieper et al. [33]	Arsenic	0.10%
	Cadmium	0.60%
	Chromium	0.00%
	Fluoride	0.40%
	Nitrate	1.30%
	Total coliform	46%
	*E*. *coli*	10%
	Copper	12%
	Lead	19%
	Aluminium	3.80%
	Chloride	0.20%
	Copper	15%
	Iron	8.00%
	Manganese	10.00%
	pH	26%
	Silver	0.00%
	Sulphate	2.40%
	TDS	10%
	Zinc	3.10%
Walker et al. [39]	Arsenic	N/A (did not present proportion who actually exceeded MAC)
Poe et al. [31]	Nitrate	18%
Lothorp et al. [51]	Aluminium	31.30%
	Arsenic	37.50%
	Iron	6.25%
	Lead	6.25%
	Antimony	6.25%
	Water Hardness	N/A
Postma et al. [34]	Total coliform	18%
	*E*. *coli*	< 1%
	Nitrates	2%
	Lead	0%
	Copper	0%
	Arsenic	6%
	Fluoride	2%
	Synthetic organic chemicals	6%
Slotnick et al. [69]	Arsenic	25.30%
Hexemer et al. [72]	Bacteriological (*E*. *coli* and total coliforms)	15.40%
	Nitrates	25.30%
Tabbot et al. [71]	Total coliform	14%
	Nitrates	58%
	Volatile organic compounds	26%
	Hardness	28%
Swistock et al. [73]	Total coliform	33%
	*E*. *coli*	14%
	pH	20%
	Lead	12%
	Nitrates	2%
	Arsenic	2%
	Triazane	< 1%
Strauss et al. [36]	Total coliform	17.10%
	*E*. c*oli*	9.50%
Yu et al. [76]	Arsenic	4.50%
Kite-Powell et al. [70]	Nitrates	55%* (during the two periods of data collection)
Lewandowski et al. [32]	Nitrates	10%* (based on well type)
Levallois et al. [42]	Nitrates	6%

Knowledge of the level of contaminants within well water was an important factor when well owners had to decide on treatment systems to use in their wells. For example, half of respondents indicated they would begin treating or finding other water sources before the concentration reached the MAC 10 mg/l of nitrates in their well water. Interestingly, a similar proportion of participants indicated that they would wait until the concentration of nitrates in their water was > 10 mg/l or higher [32]. However, the authors noted that stated intentions differed from the actual responses with only 21.9% opting to use a treatment system and about 25% opting to switch to bottled water and drilling a new well upon learning of exceedances. Flanagan et al. (2015) found that about 43% of well water owners installed water treatments with a further 30% seeking alternative water sources after being informed of exceedances in the MAC of arsenic in their well water. Therefore, even though some well owners knew their water wells exceeded the MAC for nitrates, their decision to adopt treatment or use alternative water sources may have been influenced by the perceived risk of the contaminant towards their health. These findings demonstrate the complexity in how the appraisal of the risks posed by contaminants may be highly subjective to individuals.

### Perceived risk

Individuals respond to hazards they perceive within their environment. Risk perception is defined as the subjective judgement that an individual makes about the characteristics and severity of a risk [38]. In order for an individual to make a decision on whether or not to use treatments or seek alternative drinking water sources, they must first identify the hazard (e.g. nitrates), evaluate the risk of contamination based on potential risk factors in their environment and their exposure to hazards (e.g. test for contamination in an area with extensive manure or fertiliser spread and understand how likely they are to be exposed to nitrate contamination) and finally they must understand the consequences of the hazard and their ability to control those consequences (e.g. know about a health risk such as methemoglobinemia and make a judgement on the severity of the methemoglobinemia towards their own health). Given there are several contaminants that may be considered hazards to well water, the process of assessing the risk of general well water contamination without a specific preidentified hazard may be problematic for well owners therefore making the decision of treatment options, whether to switch to an alternative or what and when to test for water quality more difficult [1]. Individuals were less likely to drink well water if they thought there were health risks associated with consuming water with arsenic [39]. Similarly, well water owners were less likely to drink well water if they perceived a risk in drinking well water regardless of aesthetic concerns [40, 41]. Perceived risk factors within the environment could also influence what people think of their well water quality. Participants reported proximity to livestock, proximity to septic systems, proximity to oil and gas activities, proximity to mining areas, proximity to nuclear power plants, flooding, severe runoff events, and drought as environmental risks that caused concern and motivated well owners to test their water [27, 34, 42–46]. However, the perceptions of water quality in response to environmental risk factors were indirectly mediated by actual changes in the aesthetic properties of water as some participants noted.

### Demographic factors

Demographics can influence the choices well water owners make of drinking water options. Factors such a participant’s education, income, number of years within a residence, and place of residence have been noted as important factors that influenced perceptions of water quality and the willingness to use water treatment [26, 29, 30, 47, 48]. Low education and income were more likely to result in the lack of use of well water treatment devices [26, 30]. Low education and income may also be socioeconomic factors that predispose well water owners to certain risk factors. Garcia et al. (2016) noted that residents living in underprivileged communities within New Mexico had unreliable drinking water systems, poor sanitation, and a lack of access to water testing and treatment. Despite the risk of arsenic being randomly distributed within socioeconomic groups, individuals with lower income and lower education were less likely to adopt protective behaviours such as well testing and treatment for their water wells [49]. Furthermore, psychological factors influencing testing and treatment were more prevalent among those with higher income and education. Similarly, higher education and income were positively associated with the decision to test well water quality and use water treatment devices [50, 51]. Education and income were not always associated with positive outcomes on treatment and testing. No significant association was found between education and stewardship behaviours conducted by well water owners [52]. Similarly, no significant association was found between education and income and the use of well water treatments [34, 46]. In contrast, Shaw et al. (2005) found a negative association between income, education, and the decision to use well water treatments. The number of years an individual had lived at a residence and the length of time they had used their well water also seemed to play an important role in predicting water testing and treatment behaviour. This is because well owners may get habituated to their drinking water source. Shaw et al. (2005) found that the longer an individual had lived in the household, the less likely they were to engage in well water testing behaviour. Similarly, the longer an individual had lived within the household, the less likely they were to conduct a water quality test within the last 5 years and the less likely they were willing to submit a water quality test [18]. However, some studies failed to find a significant association between the number of years lived within the home and water treatment practices [51].

Age and gender have also been explored as demographic variables that can influence perceptions of private water quality. Evidence to show associations between age and gender on perceptions of well water quality has been sparse. Age and gender did not predict well water testing behaviour among well owners [50]. With respect to gender, a significant association has been found between women and the use of well water treatment systems. This is because the presence of children within a household may be identified as a reason for concern among parents and a reason for well water owners to choose alternative drinking water sources [44, 45, 50, 53, 54].

### Past experience

The role of past experience with water quality issues is important. Past negative experiences with well water quality were found to predict well water testing behaviour [55]. These experiences were either on the individual well or within the well owners’ community. Learning of water contamination among neighbours and experiencing unexplained gastrointestinal illness were noted as motivators for individuals to conduct well water testing [18]. Despite past negative experiences being noted to influence perception of drinking water quality, determining the validity of reported past negative experiences may be subject to recall bias among surveyed participants. Furthermore, participants may not always attribute personal health problems, such as gastrointestinal illness, to drinking water from their wells. As gastrointestinal illnesses may be underreported and deemed controllable, it may be difficult to get an accurate representation of how past negative experiences with gastrointestinal illness influence perceptions of well water quality [54, 56, 57]. For well water contaminants which do not present direct clinical symptoms and may have severe health consequences due to chronic exposure (e.g., arsenic), the role of past experience associated with negative health outcomes on perception of well water quality is difficult to determine. However, past negative experience with contamination indicated by well water testing may change the perspective of well water owners with regards to the safety of their drinking water [32].

Previous positive experience with water quality testing may also influence the likelihood of well owners testing water quality in the future. For example, well owners reported being more confident in their well water supplies and therefore less likely to test their well water quality if the result of the water quality test they had conducted in the past showed no evidence of contamination [1]. Recurrent problems with well water quality as indicated by water quality test may also cause individuals to worry more about their well water quality and therefore conduct frequent testing. For example, well owners who were identified as being high risk for arsenic contamination through water testing and who knew they were at a higher risk of arsenic contamination were more likely to conduct well water testing than individuals who were identified as low risk for arsenic contamination [49]. Similarly, well owners who had engaged in previous water testing and were aware of water quality issues were more likely to conduct routine testing [58].

### External information

The impact of external information on changing perceptions towards well water quality to promote testing or treatment has been explored. External information sources may be in the from media campaigns, educational awareness programs or from prompts given by members of the society to encourage a behaviour. The format of the information presented may by varied including pamphlets and flyers distributed by public and private water public health agencies, news items, advertisements or advisories distributed through print media, social media, television or radio, information workshops, information solicited directly from water public health agencies (e.g. through phone calls) or information gathered from social informants (e.g. neighbours and friends) [2, 29, 40, 58, 59]. Participants’ responses to educational material may be varied. Nearly 43% of participants installed water treatment systems in response to elevated arsenic levels while nearly 31% switched to alternative drinking water sources [18]. Similarly, well owners were more likely to report higher arsenic testing rates in towns that had received educational intervention programs when compared to towns that did not receive programs [60]. In response to media reports on the risk of cancer associated with arsenic exposure, only 18% of participants used mitigation strategies that were useful against arsenic despite 66% having arsenic concentrations above the MAC [29, 39, 49]. Chappells et al. (2015) found that nearly 25% of participants reported making some change to their well water management practice in response to information received from either private testing laboratories or government departments. Well owners were more likely to engage in well testing programs after the dissemination of well management information through a well stewardship program [55]. Information on well water quality in the form of testing results can also be used to change participants’ perceptions of the safety of their drinking water [31]. Interestingly, not all information campaigns may increase water well stewardship. Nearly 28% of participants did not take any well stewardship action despite being aware of elevated arsenic concentrations within their well water [18]. Therefore, exposure to media or other forms of external information may not be sufficient to modify well stewardship behaviour [58].

### Values, attitudes, and beliefs

Values, attitudes, and beliefs towards health or environmental protection may also influence well owners' willingness to adopt well stewardship practices. Well owners’ decisions to conduct stewardship practices were more influenced by whether they were satisfied with their water quality and with their knowledge and beliefs of water quality [46]. Satisficing was where well owners took on a simple belief about their water well and did not develop a strong enough knowledge base to accurately make judgements of their water quality. Furthermore, most individuals in their survey believed that it was best to not to do anything with the water well unless they had issues with it. Participants also held a wide variety of beliefs when it came to their water wells and these beliefs were not necessarily associated with negative health consequences. The role of imperfect and incomplete knowledge (e.g. wrong beliefs about aquifers and the origin of water in water wells) in the decisions of whether to adopt well stewardship practices was identified as a possible barrier [46].

### Well water infrastructure

Available infrastructure, both physical and services available for well water quality maintenance, may also influence stewardship practices. The availability of free well water testing services has often been used to encourage water quality testing among well water owners [1, 18, 19, 52]. Despite testing services being offered for free in several jurisdictions in Canada and the USA, compliance towards well water testing recommendations is usually low [19, 60]. Several barriers have been identified that inhibit well water owners from conducting regular testing. Individual well owners may face multiple barriers when deciding to go through with water testing [46, 52] (Table 4). To increase compliance towards well water testing, several studies have solicited participants’ suggestions on how to increase routine well water testing. Making pick up and drop off of water sampling kits more accessible, increasing reminders to participants to conduct water quality tests, increasing educational awareness forums, providing incentives, enforcing penalties or making well water testing mandatory through legislation have all been stated as possible measures to increase compliance towards well water testing. The availability and accessibility of infrastructure for well water treatment may also influence habits towards well water protection. However, few studies have explored the reasons behind well water owner’s choice of well water treatments

**Table 4:** Barriers to well water testing and possible solutions provided

Barrier to well water testing	Recommendation to overcome barrier	Study
Inconvenience in dropping off and picking up water sampling bottles (time to get to water test locations and hours of operation for water testing centres)	Making bottle pick up and drop off more convenient for water testing or setting up services for delivering and picking up water sampling bottles	[1, 19, 46, 52, 55]
No need to frequently conduct testing	Sending well testing reminders and making the issue of well water testing more salient to well water owners	[1, 19, 46, 52, 55, 60]
Lack of information or misinformation on water testing	Educational/information awareness programs	[1, 19]
Forgetfulness of procrastination	Sending reminders	[1, 19]
No stated reason	Educational/information awareness programs	[1, 19]
Costs	Provide cost sharing or incentives	[1, 19, 46]
No health problems attributed to well water testing or no problem perception	Provide educational/information awareness programs	[1, 19]
Use of water treatment	Education/information awareness on what treatments to use	[1, 19]
Interpretation of water quality result	Education/information awareness on what exceedances to MAC’s mean	[46, 52]

.

## Discussion

This systematic review included 52 journal articles with data collected from well water owners in Canada and the USA. Perceptions of well water quality across Canada and the USA were found to be influenced by several factors. Main factors identified through this review were organoleptic properties of water, knowledge of chemical and microbiological contaminants, perceived risk, demographic factors, past experience with water quality, external information, values, attitudes, and beliefs about water, and water infrastructure. The reliance on the organoleptic properties of water to make judgements on the safety of drinking water by private water users is profound and has been identified as a key factor in other reviews [20]. To the best of our knowledge, only two previous literature reviews [20, 61] had attempted to provide a review on factors influencing perceptions of water quality.

Well water management practices are discussed in the context of testing and/or treatments. Well water testing practices often tend to be the focus for researchers and intervention strategies [1, 19, 52, 60, 62]. Widespread adoption of well testing and compliance towards recommendations set for testing tend to be problematic for well water owners to achieve. Interventions focusing on modifying well water testing behaviour based on incentives, legislation, education or community outreach activities have had moderate success on increasing compliance towards well water testing [2, 52, 60].

Interventions based on getting well water owners to adopt well water treatment are contingent on well owners understanding contaminants and the potential health risks they may pose. However due to the variety of possible contaminants found within well water, it may be very difficult to prescribe treatment devices, unless a contaminant is identified through testing, as one device may not be effective at removing all contaminants. The use of multiple well water treatment devices may offer more protection against several contaminants; however, water testing will still need to verify well water quality and identify possible risks to a well. Therefore, educating private water users on options available for them with respect to water treatment may enable private water users make more informed decisions based on the identified risks to their private water sources. The need for more information on water treatment has been identified in previous surveys [1, 19, 59]. Information from this study will be useful in informing private water users, researchers, and educators on some of the present gaps in the literature and research areas that need to be expanded on. 

### Gaps identified

Despite the focus on well water testing, very few studies have tried to discriminate which health risks are perceived to be associated with drinking water contamination and more specifically towards individual contaminants [63]. More studies are required to address this gap in knowledge between the perception of well water quality and the potential health consequences well water owners attribute to well water contamination.

Maintenance of well water stewardship behaviour such as testing, post intervention, is also an issue that has yet to be adequately addressed. Despite the role research may play in active surveillance of well water and instigating well owners to conduct water testing during the duration of the research program, there is very little evidence that behaviour such as water testing is continued after the research programs or other intervention programs end. Future research should look into assessing if well water testing behaviour is maintained among well owners and this could be done by broadening the methods to include cohort studies and not only cross-sectional designs. Broadening active surveillance periods using research may also help in determining the period prevalence of well contamination over time and address reliability issues associated with surveys by following up on well owners’ behaviour, in addition to determining the maintenance of well water stewardship practices.

Despite the amount of research that has been conducted on well water testing behaviour, compliance towards well water testing recommendations is still considered low in many jurisdictions. Changes in technology over the last 30 years and increased internet connectivity in Canada and the USA may provide well water owners with more access to information regarding their water wells. However, a potential problem that arises is what information sources should well owners trust given that current policies in well stewardship are only recommendations. More studies need to be conducted on the quality of information provided for by interventions such as educational programs or online information. Assessing the quality of information and how it is understood by well water owners may influence the adoption of well stewardship behaviours and may be important in dealing with satisficing and complacency among well owners. Furthermore, more research needs to be conducted on sources of information private water owners have access to and the uptake of information based on its trustworthiness [46, 47].

The adoption of qualitative and mixed method designs to further study perceptions of private water quality over the last decade and the shift away from quantitative studies has helped in developing a richer understanding of the issues faced by well water owners with respect to water quality. Qualitative and mixed methods research may be more beneficial in capturing the unique personal experiences and knowledge private water owners have of their water quality. Furthermore, incorporating the voice of private water owners in research may be an important step in developing well management policy and practices that will directly tap into the needs of private water users.

Despite having identified factors that influence well owners' perceptions of well water quality, it is important to note the paucity of research on how combinations of these factors influence well stewardship behaviour. There is very little evidence to suggest that perceptions of well water quality and well stewardship practices (i.e. testing and treatment) are driven by a single factor and are more likely to be influenced by a combination of several factors. While research to date has done an adequate job of identifying factors that influence perceptions of well water quality and predictors of well water stewardship, there is a knowledge gap in how these factors interact with each other to produce the desired outcome (e.g. well testing) in well owners. For example, although external information (e.g. educational forums) may help encourage well testing, if well owners conduct a well test and have a negative test result due to the educational program, how does the past experience of having a negative well test result influence both their appraisal of susceptibility to well water contamination and their willingness to test their water in the future. More research is required on how factors that influence perceptions of water quality may act synergistically or antagonistically to influence well stewardship behaviour.

This review summarises research that has been conducted on well water owners’ perceptions of water quality over the last 30 years while identifying questions and areas that need further development in research. Policies and recommendations for well water testing, treatment, and other management practices are highly contextual to the regions; however, this study summarises the most pertinent factors driving perceptions of private water based on research that has been conducted.

### Limitations

Publication bias may have been present due to the selection of articles from peer reviewed journals. Furthermore, because we only selected articles published within the last 30 years, there may have been a time lag bias with the selection of articles [64]. Despite the search for articles and selection of articles relevant for the review being restricted to the language spoken by the authors, no systematic bias has been found in reviews published in English [65].

Although their may be relationships between education and income to private water stewardship behaviours, it was difficult to operationalise or standardise income and education variables. This was because of differences in education standards and currency between Canada and the USA, income levels within different jurisdictions, and changes to income and education levels over a 30-year period. Furthermore, it was difficult to operationalise variables such as income and education levels because of differences in the what researchers choose to operationalise as ‘low education’ and ‘low income’ within their studies.

## Conclusion

Given that perceptions of water quality among private water users are influenced by several factors, researchers, educators and policy makers should appreciate the heterogeneity and interplay of these factors when planning private water management programs or developing policies. Education and communication strategies that focus more on individual well owners and their needs, based on risks identified around their well, need to be adopted as opposed to blanket policies or programs. The use of questionnaire surveys and qualitative research to identify the needs of individual well owners may help. This is especially pertinent because of the different interacting, and sometimes confounding, factors that may motivate private water users to comply with water testing and treatment recommendations.

## Additional files

Additional file 1: **Table S1.** Search terms used and papers generated on each database with search terms. (DOCX 24 kb)

Additional file 2: **Table S2.** Data abstraction for articles included in the systematic review. (XLSX 96 kb)
