# Supplementary material for: Factors influencing perceptions of private water quality in North America: a systematic review
Source: Syst Rev. 2019 May 10;8:111. doi: 10.1186/s13643-019-1013-9 (PMC6511211; doi:10.1186/s13643-019-1013-9)
Supplement: Supplementary file 1 — Table S1. Search terms used and papers generated on each database with search terms. (DOCX 24 kb) [file 13643_2019_1013_MOESM1_ESM.docx]

**Additional file 1**

**Table S1: Search terms used and papers generated on each database with search terms**

| Keyword search terms combinations | W.O.S | Medline (OVID) | Scopus | Pubmed | Agricola | EBSCO |
| --- | --- | --- | --- | --- | --- | --- |
| Private water AND Perceptions AND Treatment AND Survey | 8 | 2 | 10 | 16 | 1 | 19 |
| Private water AND Perceptions AND Treatment AND Water Quality | 9 | 2 | 16 | 4 | 1 | 24 |
| Private water AND Perceptions AND Water Quality | 56 | 2 | 71 | 12 | 3 | 164 |
| Private water AND Perceptions AND Water Quality AND Testing | 12 | 2 | 7 | 4 | 1 | 11 |
| Private water AND Perceptions AND Treatment | 15 | 3 | 26 | 27 | 2 | 62 |
| Private water AND Perceptions AND Treatment | 6 | 3 | 26 | 26 | 2 | 137 |
| Private water AND Knowledge AND Treatment | 28 | 7 | 77 | 83 | 4 | 399 |
| Private water AND Knowledge AND Testing | 49 | 4 | 29 | 18 | 2 | 1 |
| Private water AND Knowledge AND Testing AND Treatment | 9 | 3 | 12 | 11 | 11 | 1 |
| Private water AND Perception AND Testing AND Treatment | 6 | 3 | 11 | 11 | 1146 | 3 |
| Private water and Awareness AND Testing | 15 | 1 | 12 | 7 | 563 | 130 |
| Private water and Awareness AND Treatment | 19 | 1 | 29 | 36 | 3 | 217 |
| Domestic water AND Perceptions AND Treatment AND Survey | 6 | 0 | 10 | 6 | 1 | 854 |
| Domestic water AND Perception AND Treatment | 23 | 1 | 61 | 16 | 3 | 400 |
| Domestic water AND Perception AND Treatment AND Testing | 5 | 0 | 4 | 1 | 1184 | 528 |
| Domestic water AND Knowledge AND Testing | 58 | 2 | 18 | 12 | 3 | 515 |
| Domestic water AND Knowledge AND Treatment AND Testing | 17 | 1 | 11 | 8 | 2 | 716 |
| Private water AND Information AND Treatment AND Testing | 15 | 3 | 14 | 11 | 1611 | 3213 |
| Domestic water AND Information AND Treatment | 153 | 4 | 433 | 163 | 22 | 3 |
| Domestic water AND Information AND Treatment AND Testing | 31 | 1 | 20 | 6 | 1 | 1532 |
| Domestic water AND Awareness AND Testing | 14 | 1 | 8 | 7 | 560 | 436 |
| Domestic water AND Awareness AND Treatment | 32 | 2 | 64 | 36 | 5 | 285 |
| Domestic water AND Knowledge AND Treatment | 94 | 4 | 201 | 124 | 11 | 751 |
| Household water AND Information AND Treatment AND Testing | 27 | 1 | 20 | 10 | 2 | 69 |
| Well water AND Perceptions AND Treatment | 137 | 0 | 258 | 351 | 9 | 682 |
| Well water AND Information AND Beliefs | 75 | 2 | 116 | 171 | 7 | 483 |
| Well water AND Knowledge AND Belief | 94 | 16 | 144 | 162 | 9 | 427 |
| Well water AND Knowledge AND Attitude | 154 | 15 | 404* | 248 | 18 | 882 |
| Well Water AND Awareness AND Belief | 19 | 0 | 67 | 20 | 4 | 113 |
| Well Water AND Awareness AND Attitude | 62 | 3 | 147 | 60 | 15 | 417 |
| Well Water AND Perceptions Information AND Testing | 40 | 0 | 38 | 11 | 2 | 91 |
| Well Water AND Information AND Testing AND Survey | 224 | 4 | 138 | 11 | 7 | 1704 |
| Well Water AND Information AND Treatment | 1719 | 16 | 138 | 1338 | 230 | 8603 |
| Well Water AND Information AND Treatment AND Survey | 100 | 5 | 218 | 137 | 18 | 730 |
| Drinking water AND Information AND Treatment AND Testing | 170 | 25 | 108 | 46 | 6 | 356 |
| Drinking water AND Perception AND Treatment AND Survey | 29 | 65 | 55 | 28 | 1 | 96 |
| Drinking water AND Perception AND Treatment | 110 | 7 | 292 | 28 | 4 | 565 |
| Drinking water and Perception AND Testing | 132 | 14 | 60 | 32 | 2 | 191 |
| Drinking water and Perception AND Treatment AND rural | 20 | 7 | 38 | 14 | 1 | 88 |
| Household water and Perception AND Survey | 201 | 3 | 267 | 52 | 19 | 970 |
| Household water and Perception AND Treatment | 76 | 5 | 96 | 42 | 7 | 370 |
| Drinking water AND Belief AND Treatment | 44 | 17 | 61 | 646 | 1 | 185 |
| Well water AND Survey AND Private AND Testing | 48 | 11 | 40 | 20 | 2 | 182 |
|  | 4160 | 286 | 3875 | 4072 | 5506 | 18579 |
